# *Legionella* Antibodies in a Danish Hospital Staff with Known Occupational Exposure

**DOI:** 10.1155/2009/812829

**Published:** 2009-05-26

**Authors:** M. Rudbeck, S. Viskum, K. Mølbak, S. A. Uldum

**Affiliations:** ^1^Department of Bacteriology, Mycology, and Parasitology, Statens Serum Institut, DK-2300 Copenhagen, Denmark; ^2^Department of Occupational and Environmental Medicine, Aarhus University Hospital, DK-9000 Aalborg, Denmark; ^3^Department of Epidemiology, Statens Serum Institut, DK-9000 Copenhagen, Denmark

## Abstract

Although legionnaires' disease frequently is acquired in health care institutions, little is known about the occupational risk of *Legionella* infection among health care workers. The aim of the present cross-sectional study was to analyse antibody levels among exposed hospital workers and to determine the correlation between antibodies to *Legionella* and self-reported symptoms. The study included 258 hospital employees and a reference group of 708 healthy blood donors. Hospital workers had a higher prevalence of *Legionella* antibody titres (≥1 : 128) than blood donors (odds ratio 3.4; 95% CI 2.4–4.8). Antibody levels were not higher among staff members at risk of frequent aerosol exposure than among less exposed employees. There was no consistent association between a history of influenza-like symptom complex and the presence of antibodies. The results indicate that hospital workers have a higher risk of *Legionella* infections than the general population. However, since no excess morbidity was associated with seropositivity, most *Legionella* infections may be asymptomatic.

## 1. Introduction

*Legionella* spp. are frequently present in the water systems of large buildings, and exposure to these bacteria occurs therefore regularly. Nonetheless, legionnaires' diseases (LDs), the most severe form of illness due to *Legionella* spp., seem to be a rare outcome of exposure. This has been underpinned by outbreak investigations suggesting that only 0.1–5% of persons exposed to *Legionella* develops LD. Most *Legionella* infections may be subclinical or result in an influenza-like illness (Pontiac fever). In particular, subclinical infections may be common among individuals with regular exposure to *Legionella* [1, 2]. In an outbreak of LD at a floral show, antibody levels were higher in exposed but asymptomatic exhibitors than in the general population. Health complaints differed by the workplace locations of the exhibitors but were largely independent of antibody levels [3].

Although *Legionella* has been detected by culture in up to 70% of water samples from hospitals' water distribution systems [4–8], and nosocomial LD is a well-known problem, little is known about rates of *Legionella* infections in communities and workplaces.

The aim of the present cross-sectional study was to analyse antibody levels among hospital workers with known exposure to *Legionella* and to determine the correlation between antibodies to *Legionella* and self-reported symptoms compatible with *Legionella* infection. Furthermore, we examined domestic and other environmental risk factors for seropositivity among the hospital workers.

## 2. Methods

### 2.1. Hospital Setting

The study was undertaken at a 643-bed acute-care hospital providing both general and specialised hospital care. The hospital blocks include both new and old buildings up to a hundred years old. The hospital is supplied with municipal water without chemical treatment. There have been no cooling towers functioning in the hospital area since 2001. Before 2003 there were 21 separate hot water systems with blind ends in every system. From 1998 to 2003 all hot water tanks were removed and replaced by heat exchangers. As part of measures for reducing the risk of *Legionella* infection at the hospital, the temperature of the outgoing hot water is maintained at least 60°C; whereas the circulating temperature and the temperatures at the most remote points-of-use are at least 50°C. Once a week, the temperature is increased to 67–70°C in about three hours. There is no routine monitoring of the temperatures of the water in the pipes or at the points-of-use. In spite of these precautions, six nosocomial LD cases from five departments were reported at the hospital between 1999 and 2005. The hospital has guidelines for the prevention of LD among susceptible patients, including recommendations to avoid exposure to aerosols and to use sterile water for drinking purposes, and so forth.

### 2.2. Legionella in the Water Installations

Water samples from the hospital were analysed for viable *Legionella* at Statens Serum Institut within two days of sampling. The results were recorded as the highest number of colonies confirmed as *Legionella* (CFU/litre). From each water sample with growth of *Legionella,* one to five colonies were selected and tested by *Legionella* Latex Test (Oxoid DR0800, Basingstoke, UK), by this method the isolates were divided into *L. pneumophila* serogroup 1, *L. pneumophila* serogroup 2–14, and *Legionella* spp. non-*pneumophila.* The lowest count of *Legionella* that reliably can be detected by this method is 100 CFU/litre.

In the period 1999 to 2005, 230 waters samples were analysed, and 214 (93%) were positive for *Legionella* spp. with counts up to 28 0000 CFU/litre. All departments included had positive water tests for *L. pneumophila,* and *L. pneumophila* sg 1 were found in all departments but one. The samples (74) taken in the year of the study, 2005, showed that all water distribution systems of the selected departments were positive for *Legionella* with counts up to 18 000 CFU/litre. *L. pneumophila* sg 1 was present in 14% of the samples, sg 2–14 in 60% (*L. pneumophila* sg 3 in 19%), and in 1% of the samples *Legionella* spp. (non-*pneumophila*). A month before our study we tested representative showers at the departments and at the staff changing-rooms; three of four showers at the staff changing-rooms showed low levels of *Legionella* spp.

### 2.3. Study Population

A total of 675 employees from nine different hospital departments were invited to participate in the study. The eligible employees had various risks of exposure, including showering patients, performing surgical hand wash, or using the shower of the hospital for personal purpose. A total of 258 (42%) participated. The participation rate ranged from 15% to 79% at the different departments. The sampling period was Autumn 2005.

All participants were asked to complete a questionnaire about self-reported health and relevant exposures during the past year. The questions about health status included a history of ailments such as influenza-like illness, pneumonia, common cold; health care seeking including hospitalisations and visits to general practitioners; absences from work due to illness; specific symptoms (cough, fever, malaise, stomach pain, shiver, diarrhoea, headache, myalgia, cold). Participants were requested to report symptoms only if they had persisted for at least two consecutive days in the previous year.

The questions on occupational exposures included frequency and duration of showering patients, using a shower at the hospital for personal purpose, and frequency of performing surgical hand wash. Combined hospital exposure included any frequency of showering patients, self-showering, or surgical hand wash.

The questions on nonoccupational exposures (reflecting potential environmental risk factors) included type of residence; residence built before 1970; district heating; presence of hot water tank; hot water tap-time (considered to be slow if not hot in 1/2–1 minute); closure of water distribution; closure of home; use of spa-bath; shower elsewhere than home; swimming pool; travel abroad; hotel stay in Denmark; visit to a Danish summer cottage; air-condition in private car.

Socioeconomic variables were school-education, job skills, and family income. All questions concerned exposures during the previous year.

### 2.4. Serological Methods

Blood samples from hospital workers were analysed for antibodies to *Legionella* by indirect immunofluorescence antibody test (IFAT) with plate grown and heat inactivated *L. pneumophila* serogroup (sg) 1 to 6, *L. micdadei* and *L. bozemanii* as antigens. All sera were teste against all antigens. The assay is based on the well-characterised assay described by Wilkinson et al. [9], which follows the guidelines from the Centers for Disease Control and Prevention (CDC). The assay has been adapted as described [10, 11]. The in-house IF test has recently been compared with commercial kits for detection of antibodies to *L. pneumophila,* and it was found that the in house test was at least as specific and sensitive as the commercial kits [12].The serum samples were titrated from 1 : 64 and upwards to end-point titre. A titre of ≥1 : 128 was used to define a positive antibody response to *Legionella.* National laboratory test criteria for a confirmed diagnosis of *Legionella* infection include a four-fold or greater rise in antibody titre to ≥1 : 128 in IFAT (seroconversion) to *L. pneumophila* sg 1, 3, or 6. Seroconversion to other *Legionella* antigens and positive titres (≥1 : 256) to any *Legionella* antigen are considered indicative of a recent or previous *Legionella* infection.

### 2.5. Blood Donor Population

To compare the results obtained for health care workers with the general population, we analysed blood samples collected from 308 and 400 healthy blood donors living in the two towns of Randers and Vejle, as described previously [11]. These towns are situated in the neighbour regions of the catchment area of the study hospital. In 2004, the incidences of notified LD in the two towns were 48 and 19 per million inhabitants, respectively. In 2004, the incidence of LD in the town of the study hospital was 17 per million which is within average incidence of LD in Denmark.

The serological analysis was made by the same method as described above. There was no difference in age, gender, or overall antibody distribution between the towns. Median age for the blood donors was 45 years and 57% were males.

### 2.6. Statistical Methods

Epi Data (Ver 3, Odense, Denmark) was used for data entry. Univariable analyses were performed with antibody status as the dependent variable; variables with *P* < .2 were added to the model. Based on this *P*-value, multiple logistic regression analyses were applied to determine associations with health status and risk factors, respectively, adjusted for age, gender, and current smoking. Variables of significance in the multiple analysis were reported with odds ratio (OR) and 95% confidence interval (CI). The reference group had titres below 1 : 128. The prevalence of seropositivity declined by age in a log-linear fashion and therefore age (in years) was fitted as a continuous variable. Age groups (<30, 30–39, 40–49, ≥50) were used in further analysis of age differences. Statistical analyses were done in STATA (Ver 9.2, Tex, USA).

The study was approved by the Regional Scientific Ethical Committee (VN2005/7) and the Danish Data Protection Agency.

## 3. Results

### 3.1. Comparing Antibody Levels with a Healthy Blood Donor Population

The antibody titres ≥1 : 128 for all serogroups were significantly higher in the hospital staff (45.1%) than in the donor population (22.9%) (OR 3.41, 95% CI 2.44–4.77). There was no significant difference in antibody levels for *L. pneumophila* sg 1 (OR 1.43, 95% CI 0.94–2.16).

One person only from the hospital staff was positive to *L. bozemanii,* and none was positive to *L. micdadei*.

The hospital staff had a mean age of 44 years (range 20 to 67 years) with a male/female ratio of 36/222. A total of 16.7% were smokers. There was no difference in mean age and range or smoking between the hospital staff and the healthy blood donor population [11], although nearly a quarter (22.5%) of the donors was smokers. Male/female ratio was differently, with 56.9% of the donors being males, however, among donors the antibody levels were independent of gender [11].

### 3.2. Health Status

In general, there were no marked differences in self-reported morbidity between seropositive and seronegative individuals. Persons with *Legionella* antibodies tended to report more absence from work due to illness or having had a common cold than seronegative persons reported (Table 1). Furthermore, persons who reported symptoms other than influenza-like symptoms had a lower risk of developing antibodies (OR 0.34; 95% CI 0.12–0.99), but the numbers were small (Table 1).

Based on a *P*-value of <.2, multiple regression analysis of seropositives (titre ≥1 : 128) revealed association with previous pneumonia (OR 0.29; 95% CI 0.09–0.94) and current smoking (OR 3.54; 95% 1.10–11.49). Seropositives for sg 1 (titre ≥1 : 128) were not associated with any of the health-related variables in multiple regression analyses.

Furthermore, we constructed a symptom complex of influenza-like illness (cough, fever, malaise, stomach pain, shiver, diarrhoea, headache, myalgia, cold). There were no consistent association between *Legionella* antibodies and a symptom complex of at least three (OR 1.95; 95% CI 1.00–3.78), four (OR 1.08; 95% CI 0.90–1.29), or five (OR 0.99; 95% CI 0.84–1.17) symptoms of the complex of influenza-like illness with adjustment for age, gender, and current smoking.

Finally, there were no associations between symptoms and exposures at the hospital (data not shown).

### 3.3. Risk Factors

Individuals taking showers in other places than home or having air-conditioning in private car had an increased risk of having antibodies (Table 1). The multiple regression model (seropositives with titre ≥1 : 128) of risk variables with *P* < .2 and gender, age, current smoking showed significant increase in antibodies with showering elsewhere than home (OR 1.89; 95% CI 1.08–3.31), air-conditioning in car (OR 1.99; 95% CI 1.15–3.35), and decreasing age (OR 0.97; 95% CI (0.94–0.99).

Multiple regression analysis of seropositivity to sg 1(titre ≥1 : 128) showed increased antibody levels to sg 1 when having a hot water tank at home (OR 4.49; 95% CI 1.53–13.1) and by decreasing age (OR 0.95; 95% CI 0.90–1.00) and decreased antibody levels when having had hotel stays in Denmark (OR 0.32; 95% CI 0.11–0.95). Further age analysis in age groups showed a lower prevalence in persons 50 years and above (OR 0.79; 95% CI 0.65–0.95) compared with individuals below 50 years.

Antibody levels were independent of hospital department and type of occupational exposure (data not shown). Thus, there were no significant differences in antibody level between staff members working with patients, showering patients, taking personal showers or doing surgical hand wash. There were no differences according to frequency of the exposure (almost daily to never).

## 4. Discussion

We found a higher prevalence of *Legionella* antibodies (≥1 : 128) in the hospital staff with continuous exposure from the water system than in blood donors being representative of the general health population. Antibody levels were not higher in members of the hospital staff at risk of frequent aerosol exposures from showers or surgical hand washing. We found no association between an influenza-like symptom complex and the presence of antibodies.

The epidemiology of subclinical *Legionella* infections is largely unknown, especially beyond the outbreak setting. However, outbreak investigations indicate that the antibody response in the healthy population declines with the distance to the source of the outbreak [13, 14]. An outbreak of Pontiac fever indicated coherence between attack rate and distance to source too [15]. Compared with other studies we found a high prevalence of seropositive individuals suggesting a high exposure and probably ongoing exposure at the hospital. This finding is consistent with an Italian study of a healthy hospital staff which found a high prevalence of antibodies to *L. pneumophila* sg 1–14, but only a prevalence of 3.0% for *L. pneumophila* sg 1–6 which are the serogroups (especially *L. pneumophila* sg 1) most frequently reported causing disease [16]. The distribution of the levels of *Legionella* antibody shifts to the right (higher levels) with increasing exposure in an outbreak situation [14]; a similar distribution may occur at the hospital setting with a probable higher exposure of the staff compared with the donor population. We found no specific sources of exposure at the hospital nor subgroups being at higher risk. This is in contrast to a study from Italy where dental personnel had a higher risk of antibodies compared to other hospital workers [16], possibly due to dental staff being close to aerosols. Aerosols have been shown to be able to spread over a large area outdoors [17]. We do not know if aerosols will disperse over large areas indoors, but this is conceivable. Surprisingly, a small fountain without obvious aerosols-generating capability was recently implicated as the source of an outbreak of LD [18]. This corroborates that exposure to *Legionella* arising from aerosol-generating sources at health care facilities may occur relatively far from the source. The hospital workers distance to aerosol sources at the hospital or their number of contacts with the sources had no influence on their antibody level.

The questions about exposure and health were all about conditions in the previous year. The health symptoms are common, frequent, and probably not easy to remember. This poses the problem of recall bias, but recall bias will affect both groups equally as no one is aware of having antibodies. Self-reported exposure time and frequencies of exposure at work seem to be valid and useful [19].

The association between types of symptoms and high antibody levels in some previous studies seems to be weak and inconsistent [1, 3, 20]. We found no symptoms related to high antibody level, even though single chance findings could be expected due to the large number of tested symptoms.

Our study was limited by being based on the serological analysis of single serum samples; it is well known that antibodies to *Legionella* can be detected months after an infection. Reliable serological diagnosis of a recent or current *Legionella* infection can best be done by the detection of a seroconversion, which for our IF test is defined as a fourfold rise in titre to at least 1 : 128. In addition, it can take two to several weeks before antibodies can be detected after the onset of symptoms or after exposure. A follow-up study of a staff cohort would have enabled us to detect both the changes in antibodies and the related symptoms.

We compared the hospital staff with two donor populations of two towns. We do not know to what extent the seroprevalence varies in different populations, though we found hardly any variation between our two blood donor populations in the two different towns, one with an average incidence and one with an endemic high incidence of LD, respectively [11]. We know that the incidence of LD in the study town was within the average incidence of LD in Denmark, and we therefore assume that the overall prevalence of antibodies to *Legionella* in the population in the town of our hospital was at the same level as the donor populations in the reference towns.

An inverse relation between age and seroprevalence has not been demonstrated in other studies [14, 16].

## 5. Conclusions

We investigated the staff at a hospital with an ongoing high amount of *Legionella* in the water system. We found that almost half of the staff had serological signs of *Legionella* infection, but these antibodies could not be related to specific occupational exposures or symptoms. Although this indicates that the health implications for workers at health care facilities may be limited, we do not know the health risks if a virulent *Legionella* invades the distribution system. Treatment and maintenance of water systems in healthcare to minimise the threat of *Legionella* contamination following well-described methods should therefore be a standard procedure in order not only to minimise the risk of nosocomial LD but also to reduce occupational risks.

## Figures and Tables

**Table 1:** Univariate analysis of self-reported health and risk factors in the staff with antibodies to Legionella pneumophila at a specialised hospital in Denmark, 2005. Variables with *P* < .2 in any of the two groups are included

	Titre ≥1 : 128			Titre ≥1 : 128 of L. pneumophila sg 1			Reference < 1 : 128
	(n = 116)			(n = 41)			(n=142)
	No. (%)	OR	P	No. (%)	OR	P	No. (%)
	(yes/no)	(95% CI)			(95% CI)		
Health							

Illness the previous year Absence from work due to infection in days Pneumonia during 5 years	84/115 (73)	1.58	0.090	30/41 (73)	1.58	0.238	89/141 (63)
		(0.93-2.70)			(0.73-3.40)		
	70/89 (79)	1.79	0.079	27/35 (77)	1.61	0.281	68/101 (67)
		(0.93-3.44)			(0.66-3.94)		
	10/114 (9)	0.57	0.164	7/40 (18)	1.27	0.621	20/139 (14)
		(0.26-1.28)			(0.50-3.27)		
GP visit	48/115 (42)	0.75	0.252	25/41 (61)	1.61	0.186	68/139 (49)
		(0.45-1.23)			(0.79-3.27)		
Influenza	38/116 (33)	1.67	0.72	13/41 (32)	1.60	0.237	31/137 (23)
		(0.95-2.91)			(0.74-3.46)		
Stomach ache	23/87 (26)	0.70	0.268	6/33 (18)	0.44	0.084	31/91 (34)
		(0.37-1.32)			(0.16-1.17)		
Headache	32/87 (37)	0.66	0.177	12/33 (36)	0.66	0.323	43/92 (47)
		(0.36-1.21)			(0.29-1.51)		
Myalgia	18/87 (21)	0.62	0.167	7/33 (21)	0.65	0.359	27/91 (30)
		(0.31-1.23)			(0.25-1.67)		
Common cold	64/87 (74)	1.82	0.062	26/33 (79)	2.39	0.056	55/91 (60)
		(0.97-3.44)			(0.94-6.08)		
Other symptoms	5/86 (6)	**0.34**	**0.036**	4/32 (13)	0.80	0.703	14/91 (15)
		**(0.12-0.99)**			(0.24-2.62)		

Risk factors							

Hot water tank	49/103 (48)	1.27	0.376	22/37 (59)	2.08	0.053	50/121 (41)
		(0.75-2.16)			(0.86-4.41)		
Showering elsewhere than home	58/116 (50)	1.41	0.170	21/41 (51)	1.46	0.288	58/140 (41)
		(0.86-2.32)			(0.73-2.93)		
Travel abroad	81/116 (70)	0.66	0.154	29/41 (71)	0.69	0.354	108/139 (78)
		(0.38-1.17)			(0.31-1.50)		
Hotel stay in Denmark	43/116 (37)	0.75	0.262	12/41 (29)	0.52	0.079	62/141(44)
		(0.50-1.24)			(0.25-1.10)		
Air-condition in private car	**61/114 (54)**	**1.69**	**0.040**	22/41 (54)	1.72	0.130	56/138 (41)
		**(1.02-2.78)**			(0.85-3.46)		
Job skills less-/ more than 3 years job education	22/100 (22)	1.75	0.067	10/37 (27)	1.32	0.505	40/121 (33)
		(0.96-3.21)			(0.58-2.98)		
Current smoking	22/116 (19)	1.34	0.385	9/41(22)	1.62	0.289	21/142 (15)
		(0.69-2.58)			(0.68-3.88)		
Male/female	14/116 (12)	1.39	0.347	6/41 (15)	1.02	0.967	21/141 (15)
		(0.69-2.79)			(0.38-2.73)		
Age (continuous per year)	—	**0.97**	**0.006**	—	0.98	0.258	—
		**(0.94-0.99)**			(0.95-1.01)		
